# The Key Role of Mitochondrial Function in Health and Disease

**DOI:** 10.3390/antiox12040782

**Published:** 2023-03-23

**Authors:** Iñigo San-Millán

**Affiliations:** 1 Department of Human Physiology and Nutrition, University of Colorado, Colorado Springs, CO 80198, USA; isanmill@uccs.edu or inigo.sanmillan@cuanschutz.edu; 2 Department of Medicine, Division of Endocrinology, Metabolism and Diabetes, University of Colorado Anschutz Medical Campus, Aurora, CO 80045, USA; 3 Department of Medicine, Division of Medical Oncology, University of Colorado Anschutz Medical Campus, Aurora, CO 80045, USA

**Keywords:** mitochondrial dysfunction, cellular bioenergetics, diabetes, cardiovascular disease, cancer, Alzheimer’s disease, metabolic flexibility, exercise

## Abstract

The role of mitochondrial function in health and disease has become increasingly recognized, particularly in the last two decades. Mitochondrial dysfunction as well as disruptions of cellular bioenergetics have been shown to be ubiquitous in some of the most prevalent diseases in our society, such as type 2 diabetes, cardiovascular disease, metabolic syndrome, cancer, and Alzheimer’s disease. However, the etiology and pathogenesis of mitochondrial dysfunction in multiple diseases have yet to be elucidated, making it one of the most significant medical challenges in our history. However, the rapid advances in our knowledge of cellular metabolism coupled with the novel understanding at the molecular and genetic levels show tremendous promise to one day elucidate the mysteries of this ancient organelle in order to treat it therapeutically when needed. Mitochondrial DNA mutations, infections, aging, and a lack of physical activity have been identified to be major players in mitochondrial dysfunction in multiple diseases. This review examines the complexities of mitochondrial function, whose ancient incorporation into eukaryotic cells for energy purposes was key for the survival and creation of new species. Among these complexities, the tightly intertwined bioenergetics derived from the combustion of alimentary substrates and oxygen are necessary for cellular homeostasis, including the production of reactive oxygen species. This review discusses different etiological mechanisms by which mitochondria could become dysregulated, determining the fate of multiple tissues and organs and being a protagonist in the pathogenesis of many non–communicable diseases. Finally, physical activity is a canonical evolutionary characteristic of humans that remains embedded in our genes. The normalization of a lack of physical activity in our modern society has led to the perception that exercise is an “intervention”. However, physical activity remains the modus vivendi engrained in our genes and being sedentary has been the real intervention and collateral effect of modern societies. It is well known that a lack of physical activity leads to mitochondrial dysfunction and, hence, it probably becomes a major etiological factor of many non–communicable diseases affecting modern societies. Since physical activity remains the only stimulus we know that can improve and maintain mitochondrial function, a significant emphasis on exercise promotion should be imperative in order to prevent multiple diseases. Finally, in populations with chronic diseases where mitochondrial dysfunction is involved, an individualized exercise prescription should be crucial for the “metabolic rehabilitation” of many patients. From lessons learned from elite athletes (the perfect human machines), it is possible to translate and apply multiple concepts to the betterment of populations with chronic diseases.

## 1. Introduction

The role of mitochondrial function in health and disease has become increasingly popular, especially in the past two decades. It is known that the dysregulation of mitochondrial function and cellular bioenergetics are hallmarks of many diseases, such as type 2 diabetes (T2D), cardiovascular disease (CVD), metabolic syndrome, cancer, and Alzheimer’s disease (AD) [1,2,3,4,5,6]. Although mitochondrial dysfunction is ubiquitous to many non–communicable diseases (NCDs), the etiology and pathogenesis of mitochondrial dysfunction remain elusive and the subject of important biomedical research nowadays as one of the most significant medical challenges in our history. 

The energy production of an individual is based on the metabolic demand and metabolic efficiency during exercise, resting, and fasting and in a postprandial state. Cellular bioenergetics are quite complex and tightly intertwined with the purpose of producing the necessary energy for cellular survival as well as achieving cellular homeostasis. Mitochondria are the main cellular organelles in charge of energy production and play a pivotal role in the control of cellular hemostasis. Under resting conditions, fatty acids and carbohydrates should be successfully transported into mitochondria and be oxidized to Acetyl–CoA for posterior oxidation in the Kreb cycle (tricarboxylic acid cycle (TCA)) and electron transport chain (ETC) through oxidative phosphorylation (OXPHOS) in order to synthesize the key energetic compound in the human body (ATP). Mitochondria are also the production site for reactive oxidative species (ROS), which at physiological levels behave as signaling molecules needed for cellular homeostasis. Hence, mitochondrial malfunctions will impact cellular bioenergetics, cellular function, and cellular homeostasis, making mitochondria a key player in health and disease. There are multiple effectors eliciting mitochondrial dysfunction that have been recognized, including mitochondrial DNA mutations, infections, aging, and a lack of physical activity. However, the etiology of mitochondrial dysfunction in the pathogenesis of multiple diseases has yet to be elucidated. The aim of this review is to assemble multiple components involved in the role of mitochondrial function in health and disease, especially some of the most prevalent NCDs in our society.

Furthermore, the development of assessments for mitochondrial function in humans appears imperative in order to detect or diagnose mitochondrial dysfunction or decay. If signs of mitochondrial impairment or decay are detected early in life, there could be a significant window of opportunity to intervene in order to prevent diseases or the further deterioration of existing ones as well as improve multiple diseases through enhancing mitochondrial function through lifestyle interventions such as exercise. It has been known for decades that physical activity is probably the only known intervention that can improve mitochondrial function. The “exercise as medicine” concept continues to grow among health professionals as a necessity to prescribe exercise in a personalized and individualized manner, which seems imperative in the decades to come. However, an individualized exercise prescription should be crucial for the “metabolic rehabilitation” of many patients. This task remains a challenge due to the current lack of vertical and horizontal integration of medical systems, including clinicians, multiple providers, exercise specialists, and health care systems, with the proper means and infrastructures. The scientific and individualized approach to training elite athletes has been shown to be quite successful over the last several decades. Hence, the lessons learned from the work done with elite athletes can be an important “template” to apply to populations with chronic diseases in order to prescribe individualized exercise with the goal of improving mitochondrial function, disease, and overall metabolic health.

## 2. Mitochondria, the Key Aerobic Microbe for Eukaryotic Cell Evolution

Mitochondria originated about 1.5 billion years ago from a prokaryotic origin linked to archaebacterium (“archae” meaning “ancient bacteria”). According to the endosymbiotic hypothesis proposed by Dr. Lynn Margulis in 1967, eukaryotic species evolved from aerobic prokaryotic microbes (mitochondria) that were engulfed by an eukaryotic cell leading to endosymbiosis [7]. In general, through this symbiotic relationship, mitochondria provided aerobic energy to eukaryotic cells in exchange for protection. The ability of mitochondria to conduct aerobic respiration inside the host eukaryotic cell led to a fundamental change in evolution and the origins of hundreds of new genes and proteins, leading to novel metabolic characteristics of eukaryotic cells providing transformational evolutionary advantages to multiple species including animal life. Mitochondria continued to evolve within eukaryotic cells and both entities improved their symbiotic relationship of energy and protection.

On the other hand, the Romanian biologist and chemist Eugene Macovschi developed the biostructural theory [8]. According to this theory, living cells possess a related biological structure conferring on them living features through a so–called biostructure by which living matter consists of two distinct and interdependent forms: biostructure matter, which is the living matter itself, and coexistent molecular matter, which is a combination of chemicals in non–living matter [9,10,11]. The biostructure in cells forms an inseparable unit such that, according to Macovschi, only one uniform cell could be the origin of all forms of life, contradicting the endosymbiosis hypothesis.

Today, the cell nucleus contains genes encoding for about 1200 proteins involved in mitochondrial structure, membrane, and mitochondrial DNA (mtDNA) repair [12]. Nuclear DNA (nuDNA) is the key to a mitochondrion as its genome only contains 37 genes that encode 13 proteins, all of them involved in OXPHOS; hence, the symbiotic relationship with nuDNA. Mitochondria are referred to as the “powerhouses of cells” since they provide the ATP necessary for cellular functions and life. Mitochondria are found in all cells in the body except for red blood cells, which rely on aerobic glycolysis and lactate for proliferation and survival. Although it is commonly thought that mitochondria are individual organelles, in skeletal muscle they probably evolved to become interconnected in a reticulum or a network [13], most likely penetrating deep into skeletal muscles for increased bioenergetic efficiency. 

## 3. Mitochondrial Bioenergetics Are Complex and Intertwined

The oxidation of multiple fuels occurs within the matrix of mitochondria through the TCA cycle and OXPHOS. Mitochondria oxidize all major substrates derived from macronutrients: pyruvate derived from carbohydrates, fatty acids derived from fat, and amino acids derived from protein. Lactate, the obligatory byproduct of glycolysis, is also a very important fuel for mitochondria and could even be the fuel preferred by most cells [14]. Other metabolites, such as ketone bodies, are also commonly oxidized by mitochondria, especially under stress and fasting conditions. Skeletal muscle comprises the largest organ in the body and is the largest contributor to aerobic capacity through mitochondrial respiration [15]. Hence, skeletal muscle mitochondrial function is crucial for whole–body metabolic function and health.

Under normal, healthy conditions, mitochondrial bioenergetics are complex and tightly regulated for cellular homeostasis. In general, pyruvate, fatty acids, and a few amino acids are linked together upon being converted to Acetyl–CoA, which is the first step in the TCA cycle. The final step in the TCA cycle is the production of reducing equivalents of NADH and FADH2, which deliver electrons and hydrogen ions (H+) to mitochondrial complexes through the electron transport chain (ETC) in the inner mitochondrial membrane. These electrons build up a chemical gradient that drives ATP production. Hydrogen ions are pumped out from the mitochondrial matrix into the intermembrane space through mitochondrial complexes (I to IV). The large gradient of protons that accumulate in the intermediate space will force H+ back to the lower gradient in the mitochondrial matrix to generate ATP.

Even before oxidation, mitochondrial transport of multiple substrates is of key importance. In general, medium–chain fatty acids (FAs) can freely enter mitochondria, while long–chain FAs need to be transported through palmitoyltransferase–1 and 2 (CPT–1/2) located on the outer and inner mitochondrial membranes, respectively. Fatty acids are converted to Acyl–CoA, which through β–oxidation is converted to Acetyl–CoA for oxidation in the TCA. Pyruvate, on the other hand, is transported across mitochondria by the mitochondrial pyruvate carrier (MPC) and oxidized to Acetyl–CoA by pyruvate dehydrogenase (PDH). Therefore, the dysfunction of any of these elements involved in substrate transport across mitochondria can severely disrupt cellular bioenergetics and function.

Substrate kinetics and dynamics are also important in cellular bioenergetics. When there is increased glycolytic flux, such as in the case of high–intensity exercise or high CHO ingestion, pyruvate may accumulate even under fully aerobic conditions and have difficulty being transported across mitochondria and oxidized to Acetyl–CoA, leading to its reduction to lactate. This is a ubiquitous process in exercise bioenergetics where PDH and lactate dehydrogenase (LDH) enzyme kinetics as well as MPC transport kinetics are key players. The LDH–A isoform possess a higher affinity for pyruvate, therefore eliciting a higher rate of pyruvate reduction to lactate. Further, in the oxidation of 2 Glyceraldehyde–3–phosphate (G–3–P) to 1,3–Diphosphoglycerate, NAD^+^ is reduced to NADH and, under high glycolytic flux, cytosolic NAD^+^ may be depleted, leading to halted glycolysis and the disruption of the NAD^+^/NADH ratio and intracellular redox state. During this stressful cellular event, NAD^+^ is “rescued” by lactate through the reduction of pyruvate to lactate through LDH–A and the oxidation of NADH to NAD^+^ for the continuation of glycolysis and the stabilization of the cellular redox state. Furthermore, increased glycolytic flux can lead to the accumulation of Acetyl–CoA, resulting in inhibition of Malonyl Co–A, which inhibits CPT1 and, therefore, fatty acid transport across the mitochondrial membrane [16].

Lactate is a canonical component of cell biology and at the crossroads of cellular bioenergetics and intermediary metabolism. Lactate is the obligatory end product of glycolysis and behaves as a “lacthormone” [14,17] by possessing multiple endocrine, paracrine, and autocrine properties. Lactate is mainly oxidized in mitochondria through the mitochondrial lactate oxidation complex (mLOC) [18,19]. Poor mitochondrial lactate oxidation could lead to a significant dysregulation of cellular bioenergetics. Both lactate accumulation in the cytosol and exportation to the blood could have significant effects on the regulation of both fat and carbohydrate metabolism, tightly regulating the intermediary metabolism as well as having an intimate relationship with mitochondrial function. Lactatemia decreases the mRNA expression of GLUT 4 in skeletal muscles, therefore decreasing glucose uptake and oxidation [20]. Furthermore, lactate binds to the G–protein–coupled receptor (GPR81) on adipocytes, which inhibits lipolysis [21,22]. Moreover, we have recently shown that lactate decreases the activity of both CPT1 and CPT2 in neonatal rat cardiomyocytes, disrupts cardiolipin species, increases reactive oxidative species (ROS), and disrupts cellular bioenergetics by decreasing the rate of ATP production [23]. A decrease in lactate and fat oxidation (FATox) by mitochondria, as in the case of T2D or metabolic syndrome, indicates a direct relationship between lactatemia and FATox [24].

Metabolic flexibility is a term that has emerged in the last two decades and continues to evolve due to its involvement in multiple diseases. Mitochondrial flexibility is defined as the ability to respond or adapt to conditional changes in metabolic demand [25]. However, the work in metabolic flexibility probably dates back over a hundred years to the pioneering work by Harris and Benedict, who studied the metabolism of adult males and females, infants, and patients with diabetes [26,27]. Especially relevant was Benedict’s work in the 1920s and 1930s on the basal metabolism of both humans and animals [28] as well as metabolic responses to exercise. Skeletal muscle substrate utilization and bioenergetics are central to metabolic flexibility. Kelly and Mandarino elegantly demonstrated that skeletal muscle is central to the study of mitochondrial function. They observed that individuals with type 2 diabetes (T2D) and obesity showed metabolic inflexibility in postprandial conditions with altered glucose and fat oxidation [29,30,31,32]. Before the innovative studies by Kelly and Mandarino, DeFronzo and colleagues had shown that under euglycemic hypersinsulinemic clamp conditions, skeletal muscle uptakes and metabolizes ~85% of all glucose [33]. Metabolic flexibility and mitochondrial function are closely intertwined as under resting and post–prandial conditions both fat and glucose are oxidized in mitochondria via OXPHOS.

In summary, mitochondrial bioenergetics are quite complex and the studies continue to show that the disruption of mitochondrial and, in general, cellular bioenergetics is central to the pathogenesis of multiple diseases. The following decade will be decisive in unveiling further crucial aspects of mitochondrial bioenergetics as well as therapeutic targets.

## 4. Mitochondria, the Main Producers of Reactive Oxygen Species (ROS)

Oxygen consumption and reactive oxidative species (ROS) are ubiquitous to mitochondrial respiration. As electrons flow through the ETC, an estimated 0.4–4% of them leak before reaching Complex IV [34,35,36]. Hence, mitochondria are considered to be the main generators of ROS and, within mitochondria, Complex I is the main ROS–generating site [37,38]. Historically, ROS were previously thought to only cause cellular damage. However, it is well known that at physiological levels ROS generation is necessary and highly involved in the regulation of cellular homeostasis, key signaling pathways, cell proliferation, cell differentiation, cell migration, angiogenesis, and increased lifespan [39,40,41,42,43]. The process by which physiological ROS are involved in cellular homeostasis and signaling has been named “oxidative eustress” [40,44] and also mitohormesis [45,46,47,48]. Superoxide anions (O_2_^−^) and hydrogen peroxide (H_2_O_2_) are the main ROS and cells have specific mechanisms for counteracting excessive ROS production as well as scavenging free radicals through antioxidants such as superoxide dismutase (SOD), catalase (CAT), reduced glutathione (GSH), glutathione peroxidase (GPx), and glutathione reductase (GR). When ROS production exceeds the antioxidant capacity, ROS accumulate and can cause multiple cellular disruptions involved in multiple diseases [49,50,51,52,53,54,55,56,57,58]. Excess ROS production by mitochondria can cause damage to mtDNA, proteins, and lipids [35,59], which in turn can disrupt mitochondrial function and cellular homeostasis.

Although ROS production and mitochondrial dysfunction are intimately associated in multiple diseases, the mechanisms by which either primary ROS production leads to mitochondrial dysfunction or vice versa need to be elucidated. Could “faulty” mitochondrial function be responsible for excessive ROS production, or could it be the opposite? ROS generation occurs in the mitochondrial inner membrane, which is proximal to the mtDNA that depends on nuDNA for maintenance and repair. Therefore, the proximity of mtDNA to the ROS generation site makes mtDNA more vulnerable to oxidative damage [60]. A primary mitochondrial dysfunction may lead to a further increase in the generation of ROS, leading to exacerbated oxidative stress, which, in turn, may lead to further mitochondrial dysfunction in a self–perpetuating, feed–forward, and vicious cycle. As an example of the mitochondrial dysfunction and ROS balance for cellular homeostasis, deficiencies in the pyruvate dehydrogenase complex (PDC) lead to the accumulation of pyruvate and lactate, which has been shown to increase ROS levels [23] and decrease the antioxidant capacity [61]. On the other hand, lactate is an important signaling molecule as it stimulates a modest amount of ROS production, which elicits an antioxidant response for pro–survival cellular pathways such as PI3K/AKT and endoplasmic reticulum (ER) chaperones [62]. Moreover, different events and external effectors have been shown to elicit mitochondrial ROS generation. Tumor necrosis factor alpha (TNF–α), an inflammatory mediator, has been associated with increased ROS generation [63]. Several toxic metals, such as mercury, damage mtDNA and elicit lipid peroxidation as well as the depletion of glutathione, leading to increased ROS generation and further mitochondrial damage [64,65]. Iron–deficiency anemia can cause a decrease in the activity of Complex IV, eliciting a higher level of oxidative stress [66].

In summary, mitochondria are the main site for ROS generation and for an extended period of time it was thought that ROS production was exclusively detrimental to human cells. However, it is well known that ROS act as key signaling molecules necessary for cellular and mitochondrial homeostasis. Nevertheless, an understanding of the exact balance between homeostatic and pathological ROS production remains elusive and is currently an area in which important research efforts are being made.

## 5. Etiologies of Mitochondrial Dysfunction

**Figure 1:**
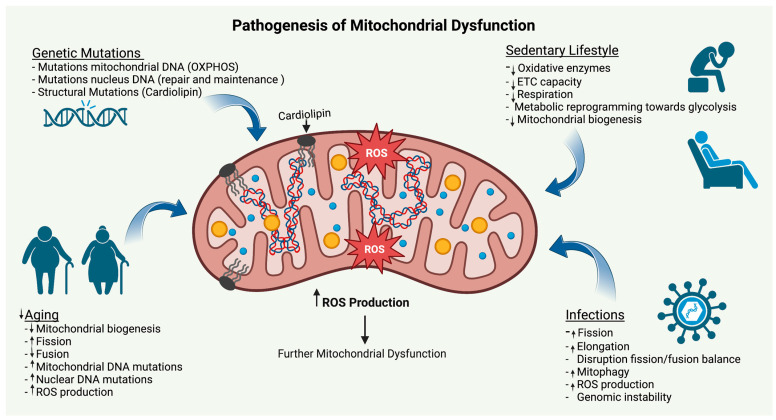
Representation of multiple effectors involved in the pathogenesis of mitochondrial dysfunction. (Up arrows indicate increased and down arrows indicate decreased).

It is important to note that the term “mitochondrial dysfunction” might not be completely appropriate. In most cases where the term “mitochondrial dysfunction” is coined, mitochondria still work but not at full or an appropriate level of potential compared with healthy states, hence the term “mitochondrial dysfunction”. However, in many situations, “decreased mitochondrial capacity” or “mitochondrial impairment” could be more appropriate. From genetic mutations to aging, infections, and a lack of physical activity, the etiology of mitochondrial dysfunction/impairment (Figure 1) is multiple and currently an important field of research due to its implications in health and disease.

### 5.1. Genetic Mutations

As previously mentioned, the mitochondrial genome contains 37 genes that encode for 13 proteins, all of which are involved in OXPHOS. Therefore, any mutation of those 13 genes could result in a significant disruption of mitochondrial function and cellular bioenergetics. The incidence of inherited mitochondrial mutations is considered to be quite rare (1 in 5000 individuals). Most mutations occurring in mtDNA are mainly point mutations and deletions [67]. Other mutations occur in the nucleus, including to autosomal recessive, dominant, or X–linked mtDNA maintenance genes [68]. Most mitochondrial genetic diseases are involved in neurological disorders, including myopathy, ataxia, and neuropathy [68].

It is important to highlight the importance of the relationship between nuDNA and mtDNA. As part of the ancient “symbiotic pact” of aerobic energy for protection between eukaryotic and prokaryotic cells, nuDNA encodes all the genes necessary for mitochondrial maintenance, repair, and replication. Hence, inherited or acquired mutations of nuDNA can contribute to mitochondrial instability [69]. Unlike germline mutations, somatic mutations evolve over the life cycle of an individual and exposure to endogenous and exogenous mutagens could lead to potential errors in nuDNA repair and replication. The symbiotic genetic relationship between nuDNA and mtDNA remains largely unexplored and could confer significant insights into the etiology of multiple diseases. 

Other mutations at the mitochondrial structural level can also affect cardiolipin (CL), which is a phospholipid in the inner mitochondrial membrane that regulates multiple mitochondrial processes [70,71] and is also involved in mitochondrial dysfunction [72]. As an example, Barth syndrome (BTSH) is a rare X–linked genetic disease caused by a mutation of the tafazzin gene, encoding for phospholipid transacylase, which is necessary for CL remodeling and characterized by cardiomyopathy, skeletal myopathy, and neutropenia [72,73,74]. BTSH is a classic example of a disruption to mitochondrial bioenergetics due to genetic mutations, leading to metabolic reprograming in the heart characterized by a significant decrease in the capacity for mitochondrial oxidation of fatty acids and pyruvate (~40–60%) [75].

Cancer is another important disease characterized by decreased/impaired mitochondrial function. mtDNA is more susceptible to DNA damage than nuDNA as it has no introns, histones, or non–histone proteins and, therefore, it is continually exposed to endogenous and exogenous mutagens, ROS, and different carcinogens [76]. This vulnerability is substantial and in cancer it has been shown that mtDNA mutations are significantly higher in number than nuDNA mutations [77,78]. Vogelstein’s group was the first to decode the mitochondrial genome in tumors, where they found mtDNA mutations in seven out of ten human colorectal cancer cell lines [79]. In a study by H.C. Lee and colleagues with 20 different types of cancer in 859 patients, 66% of those cancers carried at least one somatic mtDNA mutation [80]. In cancer, the term “mitochondrial dysfunction” refers to a significant mitochondrial impairment leading to aberrant metabolic reprograming of cellular bioenergetics characterized by accelerated glucose uptake and lactate production. It was discovered by the Nobel Laureate Otto Warburg one hundred years ago [81].

### 5.2. Aging

The process of aging has been extensively studied over decades if not centuries. Aging is an inescapable biological process characterized by decreased physiological and cellular function across the body. A decrease in mitochondrial capacity has been observed with aging and is already considered a hallmark [82,83,84,85,86,87,88,89].

Briefly, damaged and aging mitochondria are controlled by the process of mitophagy, which is the internal cellular autophagy of mitochondria. Mitophagy and mitochondrial biogenesis are indispensable to the regeneration of new mitochondria and achieve a balance for mitochondrial health. Mitochondrial fission and fusion are key processes for the regeneration and maintenance of mitochondrial networks, structure, and function. In general, mitochondrial fission splits mitochondria, where the damaged structures of mitochondria are degraded through mitophagy. The healthy fragments of mitochondria are attached together by the fusion process, allowing for the regeneration of mitochondrial structure and function, including normal metabolic bioenergetics. A decrease in the mitochondrial dynamics between fission and fusion is typical of aging processes, including increased fission and decreased fusion, which can lead to metabolic changes resulting in increased glycolysis [90,91] and the metabolic reprogramming of multiple cells.

As part of the aging process and exogenous mutagens, mtDNA mutations become more frequent and could disrupt mitochondrial dynamics and bioenergetics over time. One particular mitochondrial deletion, mtDNA^4977^, accumulates in multiple organs and is highly correlated with increased O_2_ consumption [92], which is a sign of increased glycolysis and metabolic reprograming. The GEHA EU project was an ambitious project whose goal was to compare the mtDNA variability in 2200 nonagenarian Europeans and the same number of younger individuals as a control [93]. The study showed that the association with longevity was only present when mtDNA OXPHOS complexes co–occurred [93,94].

It is also well known that, in aging, ROS and mitochondrial dysfunction are highly interconnected [95]. According to the free radicals theory of aging first proposed by Harman in 1956 [96], the ETCs inside mitochondria produce intracellular ROS that elicit mitochondrial damage and eventually cellular dysfunction. Mitochondrial ROS production can also interfere with mitophagy by disrupting the balance between fusion and fission, promoting the latter and activating intrinsic apoptotic pathways [97]. However, over the last decade the emphasis on ROS production as the underlying mechanism of the pathogenesis of aging has evolved to mitochondrial bioenergetics and turnover [86], where ROS generation could be a consequence of aging and mitochondrial dysfunction instead of the primary cause of mitochondrial injury [94]. Moreover, aging elicits an accumulation of damaged mitochondria in the brain, leading to a lower degree of metabolic efficiency, producing less ATP, and increasing the production of ROS, which can result in a disruption of cellular bioenergetics triggering neurogenerative disease [98].

### 5.3. Infections

Mitochondria play an important role in regulating the immune response to infections as they trigger multiple modulators involved in the innate immune system, including the transcriptional regulation of cytokines, chemokines, and inflammasomes [99]. Multiple bacteria and viruses modulate cellular bioenergetics in order to increase their survival rate and establish a proliferative niche. One main way by which microbes hijack cellular functions is by targeting mitochondria. Bacteria–like listeria monocytogenes, helicobacter pylori, shigella flexneri, legionella pneumophila, and chlamydia trachomatis cause mitochondrial fragmentation, mainly by increasing fission [100]. Viruses also target mitochondria through different mechanisms. The Hepatitis C virus targets mitochondria by increasing mitophagy [101]. The HIV virus disrupts the mitochondrial fission–fusion balance in the brain by increasing mitochondrial fusion, causing damage to neurons [102]. PB1–F2, an Influenza A protein, is translocated into the mitochondrial inner membrane, disrupting the membrane potential and leading to mitochondrial fragmentation [103].

Of recent importance is the pandemic caused by the SARS-CoV-2 virus. Although still under investigation, it would seem that SARS-CoV-2 also targets mitochondrial function for survival and replication by downregulating OXPHOS, increasing the elongation and overproduction of ROS [104,105]. Recently, we observed both metabolic and mitochondrial dysregulation in 50 patients infected with SARS-CoV-2 and affected by post–acute sequelae of COVID–19 (PASC), referring to extreme chronic fatigue. About half of these patients had previous comorbidities, but the other half were healthy and moderately active individuals. We observed significant metabolic dysregulation with an extremely poor capacity to oxidize fatty acids and clear lactate compared with individuals with metabolic syndrome, suggesting mitochondrial dysfunction [106]. In a subsequent study deploying metabolomics, we were able to find robust signatures of mitochondrial dysfunction and impaired fatty acid metabolism in PASC [107]. While mitochondrial function is normally restored when infections cease, patients affected by SARS-CoV-2 and suffering from long–lasting effects may have a significant and long–lasting alteration to muscle mitochondrial function, which needs to be studied in more depth.

Septicemia (sepsis) due to bacterial infection can also cause metabolic and bioenergetics disruptions in multiple organs that can ultimately lead to multi–organ failure and death. Mitochondrial dysfunction in sepsis has attracted an increasing amount of attention over the last decade in order to explain the bioenergetic dysfunction of organ failure characteristic of patients with sepsis. Impaired perfusion early on in the sepsis process, increased ROS generation, hormonal alterations, and altered transcription of mitochondrial genes can significantly affect mitochondrial function during sepsis [108]. Recently, it has been shown that mitochondrial transcription factor A (TFAM), which is key in mitochondrial biogenesis, is significantly decreased in sepsis. Rahmel and colleagues have shown that intramitochondrial TFAM levels were ~80% lower compared with controls and accompanied by decreased mtDNA copy numbers and cellular ATP content [109]. This finding is relevant as many sepsis survivors suffer from “post–sepsis syndrome”, which includes neuropathies, energetic dysfunction, and muscle weakness and wasting. The metabolic derangements in sepsis survivors also include hyperglycemia, which is a risk factor for the development of T2DM post–sepsis [110] and CVD [111].

In summary, although long–term effects of viral or bacterial infections in general are rare, a decrease in mitochondrial function caused by certain infections can elicit significant metabolic dysregulation through mitochondrial dysfunction increasing the risk of metabolism–related diseases.

### 5.4. Lack of Physical Activity

Physical inactivity has been associated with multiple diseases, including cardiovascular disease, cancer, Alzheimer’s disease, type 2 diabetes, and Parkinson’s disease [112,113,114,115,116,117,118,119,120,121,122,123,124,125] In fact, low cardiorespiratory fitness is considered to be responsible for the highest percentage of all attributable fractions for all–cause mortality [126].

The effects of a lack of physical activity on mitochondrial function have been known for decades. In 1979, Houston and colleagues found a 24% decrease in a mitochondrial function surrogate, succinate dehydrogenase (SDH), after 15 days of detraining in distance runners [127]. Coyle et al. observed that 56 days of detraining elicited a 40% decrease in mitochondrial oxidative enzyme levels and a 22% increase in lactagenic enzyme lactate dehydrogenase (LDH) levels with increased blood lactate accumulation during exercise [128]. Fritzen et al. found that 4 weeks of detraining in healthy male subjects elicited a decrease of 32% in the activity of another mitochondrial function surrogate, citrate synthase (CS), and a 29–36% decrease in mitochondrial complexes I–IV [129]. Houmard and colleagues showed a decrease in CS activity of 25% with just 14 days of detraining [130]. 

Bed studies have contributed significantly to our understanding of the loss of proper mitochondrial function and metabolic flexibility [131,132]. Alibegovic and colleagues elegantly showed that 9 days of bed rest altered more than 4500 genes and downregulated 34 metabolic pathways mainly associated with mitochondrial biogenesis, function, and OXPHOS [133]. In this study, the most downregulated pathway was OXPHOS (54% of all genes involved in OXPHOS were downregulated). Further, bed rest elicited changes in the DNA methylation of the PPARGC1A gene, which encodes for PGC–1α, a master regulator of mitochondrial biogenesis. In this same study, upon retraining for four weeks, 82% of the genetic expression that was altered with bed rest was restored, showing that physical activity restores major losses in genetic expression in a relatively short period of time. Furthermore, bed rest also induces changes in substrate partitioning favoring glycolysis instead of OXPHOS with a decrease of 37% in fat oxidation and an increase of 21% in CHO in the post–absorptive state [134]. Moreover, bed rest increases insulin resistance [135,136,137,138,139], which primarily occurs in skeletal muscle [139].

Finally, as described in Section 7 (*vide infra*), there are many studies showing that physical activity can efficiently increase mitochondrial function. Hence, the levels of daily physical activity (or the lack thereof) are significantly involved in mitochondrial function, the prevention of multiple diseases, and decreasing the risk of all–cause mortality.

## 6. The Role of Mitochondrial Function in Multiple Diseases

### 6.1. Type 2 Diabetes

Type 2 diabetes has become an unstoppable epidemic affecting millions around the world and in various countries, regardless of their degree of development and sociocultural characteristics. Currently, in the United States alone, ~52% of the adult population has either pre– or type 2 diabetes [140]. China is experiencing the largest increase in T2DM in the world [141] and Europe is also experiencing a significant increase in T2DM [142]. Other parts of the world, including developing countries such as Cuba [143] and highly developed countries such as the United Arab Emirates [144], are also being affected by this epidemic.

Insulin resistance is the hallmark of T2D and central to its pathogenesis. As previously mentioned, skeletal muscle is central to the study of mitochondrial function and its relationship to the pathogenesis of T2D. Although the mechanisms remain elusive, multiple studies over the last two decades have implicated skeletal muscle mitochondrial dysfunction in the development of insulin resistance (IR) [145,146,147,148,149,150]. It is widely known that individuals with T2DM and metabolic syndrome are characterized by decreased mitochondrial content in both intermyofibrillar and subsarcolemmal skeletal muscle regions [151,152], mitochondrial oxidative enzymes [153], mitochondrial DNA, transcriptional factors and genes [154,155,156], and overall mitochondrial function [149,151,153,157,158,159,160]. Furthermore, dysregulated muscle bioenergetics are a prevalent feature in individuals with type 2 diabetes, characterized by a poor capacity to oxidize fats and carbohydrates [25,29,161,162]. The decreased capacity to oxidize both FAs and CHO in mitochondria leads to metabolic inflexibility [32,163] and metabolic reprogramming with increased reliance on cytosolic glycolysis and lactate production to generate ATP. Furthermore, a lack of mitochondrial capacity for fat oxidation may lead to an accumulation of lipids in skeletal muscle adjacent to mitochondria, which is correlated with increased diacylglycerols, sphingolipids, ceramides, and insulin resistance [164,165,166,167]. In individuals with IR, insulin signaling is disrupted, resulting in a decrease in AKT phosphorylation and the translocation of skeletal muscle glucose transporter (GLUT–4) to the sarcolemma, leading to a decrease in glucose uptake [168]. Fernandez and colleagues developed a transgenic mouse model with the dominant–negative insulin–like growth factor–I receptor (KR–IGF–IR) in skeletal muscle. Expression of KR–IGF–IR abrogated IGF–1 and insulin receptors, resulting in insulin resistance in skeletal muscle [169].

Since skeletal muscle seems to be the tissue with the highest uptake of glucose, DeFronzo and Tipathy as well as Fernendez and colleagues proposed that T2D debuts in skeletal muscle and that muscle insulin resistance is the primary mechanistic event involved in the development of T2D [169,170].

### 6.2. Cardiovascular Disease

The role of mitochondrial dysfunction in cardiovascular disease has been receiving increasing attention in recent years [171]. The heart can suffer from severe metabolic reprograming and mitochondrial dysfunction with a decrease in oxidative capacity, oxidative phosphorylation, and ATP synthesis and an increase in ROS production [172,173]. The heart is the most oxidative tissue in the body and ~50–70% of ATP is synthesized through the β–oxidation of fatty acids [174] with 30–40% derived from aerobic glycolysis. Consequently, decreased mitochondrial function of the heart could lead to a disruption of the cellular bioenergetics of cardiomyocytes through increased glycolysis as in the case of cardiac hypertrophy and heart failure [175,176,177,178,179]. Furthermore, it has been shown that cardiomyocytes of patients with coronary artery disease possess 8–2000 more mtDNA deletions than healthy patients [180], which can significantly alter mitochondrial function and increase ROS production, leading to cellular damage and a dysregulated cellular metabolism. Moreover, even a small increase in glucose metabolism as a result of mitochondrial dysfunction can lead to cardiomyocytes with metabolic inflexibility [181].

Vascular tissue is also affected by mitochondrial dysfunction. mtDNA mutations and mitochondrial damage have been correlated with atherosclerosis [182,183]. Specifically, atherosclerotic plaques are characterized by mitochondrial dysfunction and reduced mtDNA copy numbers [184]. In the process of angiogenesis, vascular endothelial cells (VECs) possess a high degree of metabolic flexibility in order to adapt to the changing microenvironment of sprouting angiogenesis [185,186]. Although the mitochondrial composition of VECs is only 2–6% of the cell volume as opposed to 32% in cardiac myocytes [187], a small percentage of the volume of mitochondria in VECs may be key to maintaining their homeostasis [187]. Because of this specific phenotype, VECs rely on glycolysis and lactate for cell proliferation and angiogenesis [188,189]. In fact, about 99% of the glucose is reduced to lactate in VECs [187] as lactate is a major regulator of vascular endothelial vascular growth factor (VEGF) [190] and hypoxia–inducible factor HIF–1 [191], which are both key processes in angiogenesis. The remaining ATP synthesis is derived from fatty acids and glutamine via OXPHOS [192]. Although minor, the role of fatty acid oxidation may be of importance to control and balance VEC proliferation as disruptions in VEC bioenergetics could lead to pathophysiological conditions, including atherosclerosis and hypertension [192]. VECs also suffer from senescence associated with intrinsic mitochondrial impairments involving mtDNA mutations, ETC dysfunctions, changes in the fission–fusion balance, excessive ROS production, and decreases in antioxidant capacity [193,194,195,196,197,198,199]. 

### 6.3. Mitochondrial Dysfunction at the Crossroads of the Connection between Type 2 Diabetes and Cardiovascular Disease

The connection between T2D and CVD has received much attention, especially over the last two decades, as a large number of patients with T2D also develop CVD and vice versa [200]. In many cases, this connection has led to the confluence of both diseases into one emerging disease: cardiometabolic disease. At this point, the connection between these two diseases is mainly epidemiological as the mechanisms behind the relationship remain elusive. As a possible hypothesis, a primary mitochondrial dysfunction in skeletal muscle could be important to understanding the connection between both diseases. A significant histological finding pertaining to skeletal fat metabolism occurs in physically fit individuals as well as in individuals with T2D, where both populations show an accumulation of intramuscular triglycerides. This phenomenon is known as the “skeletal muscle lipid paradox” [164,201] as both physically fit individuals as well as individuals with T2D are characterized by the presence of a “lipid droplet” adjacent to mitochondria. However, the presence of skeletal muscle lipid or intramyocellular lipid (IMCL) content in highly metabolically fit individuals accounts for a significant source of fat oxidation during exercise [202,203,204,205]. On the other hand, in individuals with T2D, this accumulation of fat possesses different metabolic properties and lipid profiles compared with fit individuals [167]. In the case of individuals with T2D, the composition of intramuscular triglycerides is high in ceramides [164,167,206,207], which belong to a family of lipids consisting of sphingosines, which are bioactive lipid molecules and are involved in skeletal muscle insulin resistance [164,165,167,206,208,209,210,211], and mitochondrial dysfunction [212,213,214,215]. Circulating ceramide levels are already considered to be a biomarker of insulin resistance, T2D, and CVD [216,217,218,219,220,221]. Further, in the field of CVD research, it is well known that ceramides are key players in the atherosclerotic process [184,222,223,224,225,226]. Historically, circulating ceramides have been thought to primarily originate in the liver, where they are packed in lipoprotein particles and transported to different tissues [227]. However, it could be possible that the decrease in mitochondrial fat transport and oxidation in individuals with T2D could lead to chronic muscle lipid accumulation characterized by an increase in the content of ceramides that could be released into the blood and, consequently, contribute to the atherosclerotic process. Furthermore, as a possible cross–talk and transport mechanism, it has been shown that extracellular vesicles (EVs) can contain ceramides and that skeletal muscle is very active in secreting EVs [228]. Could the skeletal muscles of people with T2D secrete EVs containing ceramides to the blood in a way that could influence the atherosclerotic process? Could other components of EVs (mRNA, microRNA, proteins, enzymes, etc.) be implicated in the dysregulation of the metabolic function of the endothelial tissue? Although the relationships between CDV and T2D are quite strong, the mechanisms behind the link between these diseases remain elusive and a significant amount of research is needed.

### 6.4. Alzheimer’s Disease, Is It the Brain’s Diabetes?

Over the past two decades, an increasing number of studies have linked T2D to AD and cognitive impairment [229,230,231,232,233,234,235,236,237,238,239,240]. It is known that individuals with T2D have a 1.5–2–fold higher risk of developing CVD compared with people without T2D [230,231]. Moreover, a study by Janson and colleagues found that 81% of patients with AD had either T2D or impaired fasting glucose [229]. The same study showed that individuals with T2D possessed a higher frequency of islet amyloids and a greater extent of islet amyloids compared with control subjects.

The hypothesis of beta amyloid plaque as being the main culprit in the etiology of AD has prevailed since the mid–1980s [241]. However, the therapeutic approaches to treating AD by targeting amyloid plaque have proven to be unsuccessful. Consequently, and due to the necessity of developing novel therapies, innovative pathways and approaches to understanding the pathogenesis of AD have emerged. Consequently, research on brain metabolism and bioenergetics has emerged and attracted a significant amount of attention over the last two decades. Glucose and lactate are the main energy substrates for the brain [14,242,243]. A metabolic characteristic of patients with AD is diminished cerebral glucose metabolism characterized by a decreased capacity to uptake and oxidize glucose, signaling dysregulated brain bioenergetics [244,245,246,247,248]. A traditional methodology for studying cerebral glucose metabolism is the use of an ^18^–F–Fluorodeoxyglucose (^18^F–FDG) PET scan. Early studies from the 1990s showed decreased glucose metabolism in AD patients despite normal blood flow [248]. Recently, Hammond and Lin proposed that glucose metabolism is a better marker for predicting AD than amyloid or tau [245]. Further, there has been a recent tendency in clinical practice to incorporate ^18^F–FDG–PET in the diagnosis and progression assessment of AD [245,249,250,251,252].

The plethora of studies showing decreased cerebral glucose metabolism in patients with AD have led multiple researchers to inevitably explore the role of IR and mitochondrial dysfunction in the pathogenesis of AD. These pronounced novel interests have shown that, indeed, two main metabolic hallmarks of patients with AD are IR and mitochondrial dysfunction [253,254,255,256,257]. Since IR and mitochondrial dysfunction are also the main hallmarks of T2D, there seems to be a metabolic connection. Hence, novel terminologies such as “type 3 diabetes”, “brain diabetes”, and “end–stage type 2” have emerged in efforts to describe the pathogenesis of AD using a metabolism–centric approach. 

Furthermore, like skeletal muscle, the brain possesses a lactate shuttle key to brain bioenergetics [243]. Although glucose historically has been thought to be the main fuel for the brain, it is now well known that lactate is a key fuel for neurons, possibly the preferred fuel for the brain [242,243,258,259], and essential for long–term memory [260,261]. In skeletal muscle, the discovery of the lactate shuttle by Dr. George Brooks was instrumental in understanding skeletal muscle glucose and intermediary metabolism [14]. Briefly, lactate is shuttled from fast to slow–twitch muscle fibers, where lactate is oxidized in the mitochondria of slow–twitch muscle fibers via the mitochondrial lactate oxidative complex (mLOC) for fuel purposes [262,263]. Like skeletal muscle, the brain possesses its own lactate shuttle, which is called the “astrocyte–neuron lactate shuttle” [243,264,265]. Astrocytes play a key metabolic role in glucose metabolism as they receive glucose from the blood as well as store glycogen and break it down to glucose. Glycolysis is the main metabolic pathway for astrocytes, where most of the pyruvate is reduced to the lactate that is exported to neurons for fuel. From lessons learned from skeletal muscle metabolism, it is possible to observe similarities in brain metabolism through intracellular and extracellular lactate dynamics associated with mitochondrial function. While in skeletal muscle lactate is shuttled from fast–twitch muscle fibers to the mitochondria of slow–twitch fibers, in the brain, lactate is shuttled from astrocytes to neurons [243,265], where lactate is oxidized in the mitochondria of neurons via pyruvate oxidation. As a possible hypothesis, a mitochondrial dysfunction in neurons might lead to reduced astrocyte–derived lactate oxidation resulting in decreased pyruvate oxidation and a disruption of neuronal bioenergetics, as is the case with skeletal muscle, which is ultimately limited not only by glucose transport but by pyruvate oxidation.

The etiology of mitochondrial dysfunction in AD patients remains largely unknown. Although the mechanisms behind the pathogenesis of AD remain elusive, novel and exciting advances in our understanding of brain metabolism have been made in the last decade, opening the door towards the generation of novel diagnostic methods and therapeutics.

### 6.5. Cancer

The lack of progress in targeting genes to cure cancer has led to the development of novel areas of research and clinical applications with exciting therapeutic possibilities. These therapies include immunotherapy and targeted therapies, particularly those that use tyrosine kinase inhibitors (TKIs). Both immunotherapy and TKIs have helped to extend the lives of and even cure disease in (as in the case of immunotherapy) millions of people. Although they have efficacy in only a relatively small number of tumors and people, we can expect that new and more efficient generations of these therapies will be developed.

As a result of the necessity of expanding our understanding of cancer, the field of cancer metabolism has experienced a strong renaissance in the last two decades due to the renewed interest in the Warburg Effect [266,267]. As previously mentioned, in 1923, the German cell physiologist and Nobel Laureate Otto Warburg discovered that cancer cells show accelerated glycolysis and produce significant amounts of lactate [81,268]. Although cancer cells increase their glucose uptake, they may not oxidize pyruvate correctly in mitochondria, reducing it to lactate. The observation of the significant amount of lactate that accumulates in cancer cells led Warburg to posit that cancer is an injury to the cellular respiratory system (mitochondria). However, one century ago, DNA and genetic mutations were not known to exist, as DNA was discovered by Watson and Crick in 1953 [269]. It is widely recognized that genetic mutations are ubiquitous to cancer, especially the overexpression of genes such as RAS, MYC, and hypoxia inducible factor 1 alpha (HIF–1–alpha) and the loss of function of the tumor suppressor factor TP53, which confers on cancer cells a selective growth advantage for aberrant cell growth and proliferation [270,271,272,273]. Hence, not all types of cancer necessarily possess a mitochondrial dysfunction. 

As mentioned above, the Warburg Effect is characterized by accelerated glycolysis and increased lactate production, which was probably what struck Warburg the most. According to the “lactagenesis hypothesis”, the exacerbated lactate production due to cancer cells observed by Warburg one hundred years ago could be the explanation for and purpose of the Warburg Effect [274]. According to this hypothesis, lactate could be a major regulator of the main elements involved carcinogenesis: angiogenesis, immune escape, metastasis, and self–sufficient metabolism. Moreover, it has been shown recently that lactate is an oncometabolite capable of regulating histone acetylation [275] as well as the expression of the main genes involved in ER^+^ breast cancer cells, including RAS, MYC, and HIF–1–alpha [191]. Since lactate is a canonical element in most cancers, the transcendental question is: why is lactate so ubiquitous to cancer metabolism? As Warburg described a century ago, an injury to mitochondria could be one possible answer. The question of whether this injury is due to a genetic etiology, a metabolic dysregulation, or both will be fundamental to answer and even crucial to finally conquering cancer in the next decade. 

As previously mentioned, it is known that many cancers have some form of mitochondrial impairment/dysfunction that could be attributable to somatic mtDNA mutations (as already observed by Vogelstein’s laboratory in the late 1990s [79]) as well as mtDNA depletion [276]. Furthermore, other authors have observed direct disruptions of the mitochondrial structure affecting cristae (cristolysis) in glioblastoma multiforme (GBM) [277], which should have devastating consequences for cellular bioenergetics and homeostasis and could possibly be a reason for the high aggressiveness of GBM.

Furthermore, as previously mentioned, mitochondria highly depend on nuDNA as it encodes for ~1200 proteins necessary to mitochondrial repair, maintenance, and biogenesis. Hence, any mutation of any of the mitochondrial nuDNA–dependent genes could lead to dysregulation of the mitochondrial bioenergetics resulting in metabolic reprogramming through decreased OXPHOS as well as increased glycolysis and lactate production, leading to carcinogenesis. The crucial symbiotic relationship between the cellular nucleus and the mitochondrion dates back ~1.5 billion years and any disruption of this relationship by either external/internal mutagens or epigenetic effectors could lead to severe consequences for cellular homeostasis. Although the enduring symbiotic relations between the nucleus and the mitochondrion remain largely unexplored in cancer, they may provide us with a better understanding of cancer.

**Figure 2:**
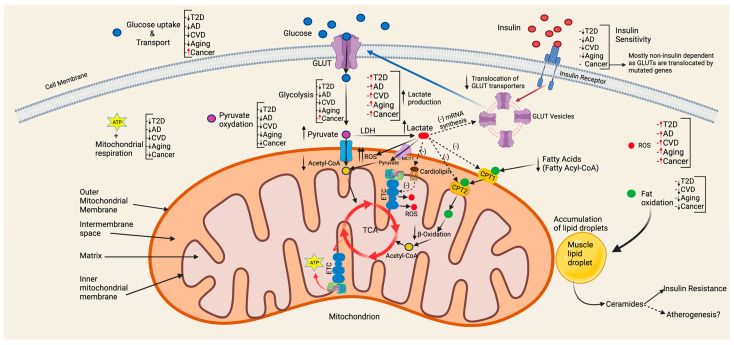
Representation of the role of mitochondrial dysfunction and disrupted bioenergetics in some of the most prevalent diseases in our society. (Red arrows indicate increased production or production in multiple diseases and black arrows indicate decreased production or function).

In summary, the implications of mitochondrial function in some of the most common NCDs are quite prevalent and central to the pathogenesis of these diseases (Figure 2). Furthermore, there is growing evidence of strong relationships between several diseases, where mitochondrial dysfunction could not only be the etiology behind the pathogenesis of these diseases but also a nexus, which, if true, would elevate medical research to horizons never before reached. Undoubtedly, the research that will be conducted during the next decade holds much promise.

## 7. Exercise, the Only Known “Medicine” for Maintaining and Improving Mitochondrial Function

It has been known for decades that exercise is the best physiological stimulus for improving mitochondrial function in skeletal muscles and possibly other organs. In this regard, we have learned many lessons from elite athletes that can be translated to multiple populations. Well–trained athletes possess the highest mitochondrial function of any humans [278,279,280,281]. The typical characteristic of elite endurance athletes is an increased capacity to oxidize fatty acids as well as carbohydrates [24,282,283,284], making them highly metabolically flexible. 

Since the late 1960s and early 1970s, multiple studies have demonstrated improvements in mitochondrial biogenesis and function after training. Twelve weeks of endurance training (5 days/week) increased the number of mitochondrial enzymes by 2–fold and the total amount of protein content by 60% [285]. Ten weeks of daily endurance training increased the mitochondrial concentration in the gastrocnemius muscle by ~30% [286] and 1 h of cycling for 4 days/week over five months at an intensity of 70–90% of the VO2max increased the oxidative capacity and glycolytic capacity by 95 and 117%, respectively [287]. Exercise can improve mitochondrial health by increasing mitochondrial content [288], increasing the transcriptional activity of mitochondrial proteins such as PGC–1α [89], and decreasing ROS production [289]. A 16–week aerobic exercise program as an intervention in both men and women showed an increase in CS and cytochrome c oxidase of 45 and 76%, respectively, as well as an increase in the expression of genes involved in mitochondrial biogenesis, such as PGC1a (55%), NRF–1 (15%), and TFAM (85%) [290].

Different studies have also used the model of training followed by detraining in order to measure muscle and mitochondrial plasticity and as a stimulus for physical activity and detraining. Moore and colleagues observed an increase of ~38% in CS activity in sedentary subjects after 7 weeks of training followed by a decrease of ~25% in CS activity and an increase of ~10% in the respiratory exchange ratio (RER) after 3 weeks of detraining, reflecting a decrease in mitochondrial oxidative capacity and flexibility [291]. Klausen et al. observed an increase of 30–40% in SDH and mitochondrial cytochrome c oxidase (COX) after 8 weeks of training, followed by a decrease to basal levels after 8 weeks of detraining [292]. Wibom et al. found an increase of 70% in the mitochondrial ATP production rate after 6 weeks of training followed by a decrease to between 12 and 28% after 3 weeks of detraining [293].

Regarding the beneficial effects of exercise on mitochondrial function in multiple diseases, there are multiple studies demonstrating the benefits of exercise on mitochondrial function. For example, a 16–week aerobic training program in sedentary, overweight/obese individuals resulted in a significant increase in mitochondria (76%) in the myofiber volume accompanied by improvements in insulin resistance that were highly correlated with mitochondrial size and content (r = 0.88 and 0.72, respectively, *p* < 0.01) [294]. Toledo and colleagues also showed that diet and weight loss alone are insufficient to stimulate the mitochondrial capacity in skeletal muscle compared with diet plus exercise [295]. In this study, both groups showed similar improvements in insulin resistance but the exercise group was the only one in which improvements in mitochondrial density, cardiolipin content, and ETC were observed. The same group of researchers showed that, in individuals with T2D, a moderate–intensity exercise program for 4 months elicited significant increases in mitochondrial density (67%), cardiolipin (55%), and mitochondrial oxidative enzymes and improved glycemic and metabolic flexibility [296]. In diabetic mice, eight weeks of aerobic exercise significantly improved the expression of mitofusin–2 (Mf2n), which improves fusion, increases the expression of the mitochondrial transcription factor PGC–1α for mitochondrial biogenesis, increases overall mitochondrial respiration, and decreases IR and ROS production [297].

In patients with mitochondrial myopathies, due to mtDNA mutations, endurance exercise has been shown to elicit significant improvements in mitochondrial function. Taivassalo and colleagues elegantly showed that 14 weeks of endurance training significantly increased the mitochondrial oxidative capacity, with increases in CS activity (~50%), SDH activity (~40%), and Complex IV (~25%) and a decrease in blood lactate accumulation (*p* < 0.05) [298,299].

In CVD, exercise is known to improve cardiomyocyte, muscle, and platelet mitochondrial biogenesis, the oxidative capacity, and the antioxidant capacity [300,301]. In patients with chronic heart failure, a six–month exercise program improved the total volume density of mitochondria by 19% [302] and the surface density of mitochondrial cristae by 43% [303]. Exercise has also been shown to significantly improve OXPHOS in the platelets of patients with stroke [304] and with peripheral artery disease [305]. Furthermore, exercise was shown to inhibit the pathological mitochondrial remodeling in rats with myocardial infarction (MI) by improving mitochondrial fusion and decreasing mitochondrial fission [306]. Furthermore, eight weeks of exercise post–MI improved the mitochondrial O2 consumption, bioenergetics, and oxidative capacity in mice [307]. 

Exercise can also improve the mitochondrial function and biogenesis in the brain as well as cognitive function [308,309], which opens an exciting door of opportunity to further understand the mechanisms behind the pathogenesis of AD and to improve the therapeutics against this disease. Therefore, we should stress the importance of physical activity not just for the prevention of T2D but possibly for mitigating the severity of and risks associated with AD as has already been shown in [310,311,312,313,314,315].

In aging, some studies have obtained promising results. Sustained endurance training over time is also quite effective at maintaining mitochondrial function and flexibility in aging populations. Dubé et al. showed that the muscle oxidative capacity, metabolic flexibility, and insulin sensitivity in older endurance–trained master athletes (average age, 65 years old) were similar to those of young recreational athletes (average age, 28 years old) [316]. Another recent study comparing the effects of exercise between elderly (average, 80 years old) and young (average age, 24 years old) cohorts found that 6 weeks of aerobic exercise increased CS activity by 31% in elderly individuals and by 45% in younger individuals. Complex I, II, III, and IV increased in both groups by between 51 and 163%. The study found that both elderly individuals and younger individuals have the capacity to improve their mitochondrial function after 6 weeks of aerobic training [317].

In summary, there are many studies demonstrating the benefits of exercise on mitochondrial function in many types of populations, including populations with chronic diseases. However, if we consider exercise to be a therapy that we can use to improve mitochondrial and metabolic function, it is essential to optimize and individualize the dose and duration of the exercise that is prescribed [318]. Over the last decade, this is an area where we have gained a wealth of knowledge by working with elite athletes to whom prescribing the right training regime is key to improving athletic performance. Translating this knowledge to populations with chronic diseases is a challenge due to the lack of vertical and horizontal integration of medical systems, including clinicians, multiple providers, exercise specialists, and health care systems, with the proper means and infrastructures. However, all stakeholders should (must) be able to materialize this multidisciplinary partnership in order to achieve proper and individualized exercise prescription programs as exercise continues to be the most important intervention that is known to improve mitochondrial function, metabolic flexibility, and, thus, metabolic health. 

## 8. Assessment of Mitochondrial and Metabolic Function in the Clinical Setting

Historically, the assessment of mitochondrial respiration and function has focused on the measurement of relevant oxidative enzymes involved in OXPHOS. CS and SDH have been traditionally used in multiple studies as surrogates for mitochondrial function and content [319]. More modern technologies have been developed to measure mitochondrial respiration and substrate utilization in skeletal muscle through two predominant techniques: the Oroboros and Seahorse technologies [320,321,322,323]. These modern techniques, as well as the traditional ones, require muscle biopsies or cell cultures, which are not feasible to obtain on a large scale in humans in order to assess mitochondrial function and respiration. Non–invasive techniques based on nuclear magnetic resonance (NMR) and magnetic resonance spectroscopy (MRS) techniques have become popular for research purposes as a valid way to assess mitochondrial respiration in vivo [324,325,326,327]. However, the application of these new techniques to the general population would be very costly and infeasible.

**Figure 3:**
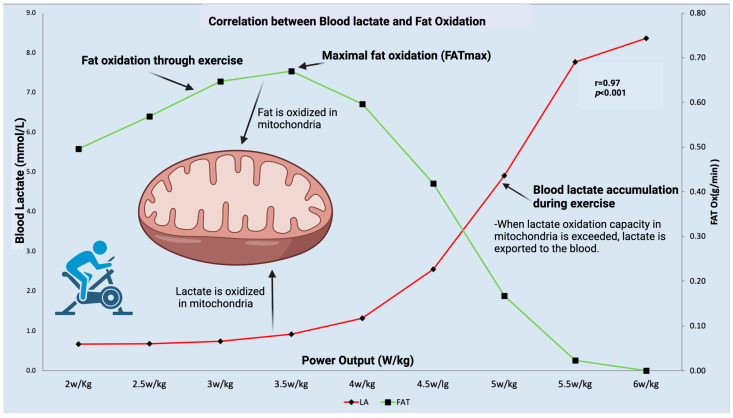
Example of a metabolic test used to indirectly measure mitochondrial capacity during exercise through measuring FATox and blood lactate levels (modified from San–Millán and Brooks, 2018) [24].

**Figure 4:**
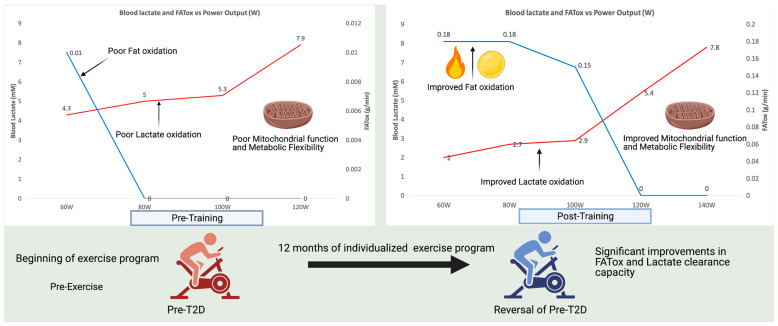
Example of the metabolic improvements of an individualized prescription exercise program on a patient diagnosed with pre–type 2 diabetes before and 12 months after completion of the exercise program. After the individualized exercise program was completed, there was a significant improvement in lactate clearance capacity and FATox, indicating an improvement in mitochondrial function (Source: San–Millan’s laboratory).

Recently, we proposed a novel and simple methodology for indirectly measuring mitochondrial function and metabolic flexibility that can be performed on a large scale in an ambulatory manner [24]. Our methodology is based on the combination of measuring fat oxidation through indirect calorimetry using stoichiometric equations and the measurement of blood lactate levels during exercise, both important mitochondrial substrates. The concept is similar to cardiology stress tests where the heart is stressed through exercise in order to measure its activity and detect pathologies. Through our methodology, we use similar protocols with incremental exercise stages in order to stress the mitochondrial capacity and detect changes in mitochondrial and muscle bioenergetics. As shown in Figure 3, during exercise, both fat oxidation and lactate are oxidized in mitochondria as they are important mitochondrial substrates. A decrease in fat oxidation capacity and an increase in blood lactate during exercise could indicate decreased mitochondrial function. We have recently applied this methodology to indirectly assess mitochondrial and metabolic function in long–COVID–19 patients (PASC) as described earlier [106] and posteriorly confirm the results with metabolomics analyses [107]. Furthermore, this methodology can be used to extract individual training zones in order to structure an individualized exercise program for a wide range of populations. Figure 4 shows an example of a person diagnosed with pre–T2D who, after one year of individualized endurance exercise, improved their lactate clearance capacity and fat oxidation significantly and was able to reverse pre–T2D.

As novel techniques arise, especially in the field of wearable biosensors, the possibilities for measuring mitochondrial function in a continuous form will become much more accurate and available to the general population as well as to clinicians. These soon–to–arrive advances in biometrics could be transformational in terms of monitoring an individual’s metabolic health status and prescribing individualized exercise programs destined, along with proper nutrition, to improve metabolic health.

## 9. Summary and Future Directions

Mitochondrial function is key in health and disease. Any effect on mitochondrial function can result in the disruption of cellular bioenergetics and the development of different pathologies. The etiology of the pathogenesis of mitochondrial disruption/impairment is diverse and multifaceted, although sedentarism seems to be a major factor. Skeletal muscle plays an instrumental role in mitochondrial function in both health and disease as it is the most bioenergetically active organ with the highest mitochondrial content. Although mitochondrial dysfunction has traditionally been linked to type 2 diabetes, it is also a hallmark of multiple diseases, including cardiovascular disease, cancer, and Alzheimer’s disease. A deeper understanding of the mechanisms behind mitochondrial function and the disruption of cellular bioenergetics in multiple diseases appears to be a major medical challenge in our history and would result in the discovery of novel biomarkers for earlier detection as well as targeted therapeutics.

In the meantime, exercise continues to be the only known stimulus for the maintenance and improvement of mitochondrial biogenesis and function. However, if exercise is prescribed as a therapeutic treatment to improve mitochondrial function (“exercise as medicine”), the correct exercise prescription will be key, as in any other therapy. In order to provide the right therapeutic benefits, individualization of the exercise prescription should be performed in order to target cellular and metabolic adaptations. This individualization of the exercise prescription has existed for decades in the world of sports performance and there are many lessons that have been learned from working with elite athletes that can be translated to exercise prescriptions for multiple populations. This task is a challenge as it involves the integration of multiple clinical providers, exercise specialists, and healthcare systems. However, this effort should be a priority in every society in order to improve our population’s health, decrease mortality rates, increase longevity, and decrease the unsustainable social and economic burden imposed by the non–communicable diseases that most countries in the world will face in the coming decades.
